# Australian Implementation of the Teaching Personal and Social Responsibility Pedagogical Model in Physical Education: Protocol for a Nonrandomized Controlled Trial

**DOI:** 10.2196/67924

**Published:** 2025-09-03

**Authors:** Sonja Maric, Wayne Cotton, Louisa Peralta, Dean Dudley

**Affiliations:** 1 Faculty of Arts and Social Science University of Sydney Sydney Australia; 2 Macquarie School of Education Faculty of Arts Macquarie University Macquarie Park Australia; 3 Centre of Educational Measurement and Assessment The University of Sydney Sydney Australia; 4 School of Human Movement and Nutrition Sciences The University of Queensland Brisbane Australia

**Keywords:** teaching personal and social responsibility, social, physical education, intervention, high school

## Abstract

**Background:**

Interventions targeting the psychomotor domain of learning have been the most dominant in the physical education (PE) discipline. However, as PE also incorporates a holistic perspective of student development, a gap has emerged where interventions targeting social outcomes are not frequently analysed. Social outcomes have been of particular interest for interventions conducted in PE.

**Objective:**

This study will evaluate the impact of an intervention designed to enhance social outcomes among Australian school students. The intervention will be shaped by the teaching personal and social responsibility (TPSR) framework. This framework has lacked representation within the Australian context, creating a gap and an opportunity to explore its impact on the social behaviors of Australian students.

**Methods:**

This study will use a nonrandomized controlled trial design with a qualitative evaluation to assess feasibility and acceptability. Data will be collected through student surveys, student focus groups, and teacher reflection journals. Two schools in New South Wales, Australia, will participate by implementing the TPSR framework in their programs. Schools will include classes in stages 3 and 4 of the intervention, involving approximately 90 students from each school. Both schools are located in Sydney, New South Wales: one is a government high school, and the other is an independent K-12 school. To address feasibility and accessibility concerns, the intervention will be delivered during regularly scheduled PE classes. It will be facilitated by students’ regular PE teachers, who will also participate in ongoing professional development and be involved in a co-design process with the research team. Primary outcomes include reporting on personal responsibility, social responsibility, and leadership behaviors, measured using two tools: (1) the Tool for Assessing Responsibility-Based Education and (2) the Personal and Social Responsibility Questionnaire. A secondary outcome, enjoyment, will be assessed using the Physical Activity Enjoyment Scale–Physical Education version. Feasibility and accessibility will also be examined as secondary outcomes.

**Results:**

This intervention study will demonstrate the impact of TPSR on social outcomes among Australian school participants. Students will also receive a briefing, and parental permission will be sought before intervention commencement. Students will be involved in the intervention for the duration of term 3 (10 weeks).

**Conclusions:**

This protocol paper outlines the approach to implementing and evaluating the TPSR intervention among Australian school participants. One anticipated strength of the intervention is the ability to apply the framework to different settings, including schools, school sport, and community organizations. Longitudinal follow-up assessment will be critical in determining the long-term impact of the intervention on behavioral changes among participants.

**Trial Registration:**

Australian New Zealand Clinical Trials Registry ACTRN12624000858594p; anzctr.org.au/Trial/Registration/TrialReview.aspx?id=387883

**International Registered Report Identifier (IRRID):**

PRR1-10.2196/67924

## Introduction

### Background

The aim of physical education (PE) lessons is to establish lifelong habits and behaviors that lead to positive health outcomes [1]. Benefits of youth participation in PE lessons include improvements in cognitive development and learning [2]; social development [3]; and a range of physiological benefits such as improved coordination, balance, weight loss, and cardiorespiratory fitness [4]. Another benefit of participation in PE lessons is access to subject matter experts such as PE teachers who can correct movement, give feedback, and influence engagement and participation [5]. However, the literature suggests that young people become disengaged in PE as they get older, which may limit the achievement of a range of cognitive, affective, social, and psychomotor outcomes [6]. To remedy this decline, emerging literature suggests that a way to re-engage students in PE may be through an emphasis on interventions with outcomes that enhance student achievement across a range of learning domains [7].

PE interventions conducted in schools have been a leading method used by researchers to analyze emerging trends in the health of young people [4,7,8]. Interventions can be categorized as psychomotor, affective, social, and cognitive, with the psychomotor domain being the most dominant in PE-based research and interventions [7]. This is evidenced by a systematic review conducted by Dudley et al [7], who found that psychomotor interventions embedded in PE settings made up 38% of the included studies. The authors suggested that future research should target other aspects of learning, such as confidence and competence, to further support young people’s development and prepare them for adulthood [7]. This argument is also supported by the Australian Curriculum: Health and Physical Education (AC: HPE) as well as its statewide equivalent, the New South Wales (NSW) K-10 personal development, health, and physical education (PDHPE) syllabus, both of which have made amendments to include a stronger emphasis on social outcomes (eg, developing empathy, relationship skills, and connectedness) in conjunction with psychomotor outcomes, which have always been prioritized in PE lessons [9]. The recently revised NSW PDHPE syllabus also features a specific strand targeting social outcomes—respectful relationships—with the sole focus of addressing and strengthening social behaviors of students [9].

Interventions targeting social outcomes in PE are not as prevalent as those targeting psychomotor outcomes, but existing studies, which are scattered within the literature, have demonstrated positive findings [10-12]. In the systematic review by Dudley et al [7], which reported on PE interventions and a range of outcomes, only 25 of the 135 included studies assessed the impact of PE on social outcomes. The study by Dudley et al [7] is important because it not only highlights emerging studies on previously underrepresented interventions targeting social outcomes but also demonstrates the gap in the literature and an opportunity for further research.

Furthermore, interventions targeting social outcomes are reported to have strong associations between student motivation and the development of autonomy through PE lessons [13]. Research suggests that students who develop skills such as leadership and responsibility behaviors experience smoother transitions from school to adult life [14]. Other research has found that student self-esteem and physical self-concept were improved through involvement in interventions targeting social outcomes [15]. However, a criticism of social interventions has been the issue of transfer, that is, whether positive effects last beyond the intervention or are merely a result of a novelty effect, which has been noted in studies featuring PE pedagogical models such as the sport education model and teaching games for understanding. To counter this criticism, the teaching personal and social responsibility (TPSR) framework has been chosen, as it has a domain dedicated to promoting transfer [16]. This framework has been used in the PE context to improve the social skills of participants and may be able to address the gap in the literature by targeting social outcomes with lasting benefits [17,18]. The sport education model and the teaching games for understanding model have been used in interventions targeting social outcomes in a variety of contexts; however, “transfer” underpins all other levels of the TPSR framework and aims to address social outcomes as a life skill, rather than as a tool for PE class [19]. Furthermore, TPSR differs from other social frameworks, as its levels are more fluid and nonhierarchical, allowing students to move in and out of the TPSR domains, rather than following a step-by-step process of development [19].

TPSR is a pedagogical framework designed by Hellison to engage students with low socioeconomic status (SES) from urban communities in PE lessons [10,16]. One of the main goals of the TPSR framework is to encourage students to apply the skills learned throughout the intervention to other key learning areas, as well as life outside the school setting [16]. TPSR aims to promote student autonomy and teach students to take responsibility for their learning [17]. The framework aims to improve skills such as empathy and leadership as well as developing and maintaining relationships, which strongly mirrors the goals and rationale of the NSW PDHPE K-10 syllabus and the AC: HPE [9,16,20]. The framework has been constructed using five levels: (1) respect for the rights and feelings of others, (2) effort and teamwork, (3) self-direction and goal setting, (4) helping and leadership, and (5) transfer [16,17]. There are also four themes for each program—(1) a strong teacher-student relationship, (2) empowering students, (3) integrating responsibility into physical activity, and (4) promoting the transfer of responsibility—which align strongly with the AC: HPE and the NSW K-10 PDHPE syllabus [9,16,17,20]. Due to a strong link between the TPSR framework and the Australian curricula, future interventions should aim to plan, implement, and test the effect of TPSR in Australian schools. Australian schools are a desirable setting for interventions using TPSR, given the similarities to the environments identified by Hellison [21] in his work with disengaged students from low-SES backgrounds. Furthermore, schools in the western suburbs of Sydney serve a high proportion of students with low SES and have noted difficulty with engagement and integration from both student and teacher perspectives [22,23]. However, TPSR implementation in Australian schools has seldom been reported, and interventions using social frameworks in PE lessons are scarce.

International research using the TPSR framework has shown promising findings [12,18,24,25]. In a systematic review, a Turkish study found that TPSR-based interventions improved students’ efficacy as well as interpersonal interactions and emotional regulation when dealing with other students and adults and in contexts where they were required to recognize and express emotion [18]. Similarly, research from Taiwan found that adherence to the TPSR framework in PE settings improved the self-efficacy and efficacy-like behaviors of middle school students [24]. A study in the United States found that students’ socially responsible behaviors, such as emotional regulation, problem-solving, and the promotion of social norms, improved as a result of TPSR-based interventions [25]. TPRS has further demonstrated an increase in motivation for engaging in PE classes, with research in Spain demonstrating that students had more positive perceptions of their athleticism as well as showed improvements in leadership and decision-making skills as a result of participation in the intervention [12]. International literature demonstrates a clear improvement in many social outcomes due to participation in interventions that apply the TPRS framework. In addition, a notable point of difference between TPSR and other social frameworks is the transfer domain, which could be particularly important when implementing a TPSR intervention in the Australian context.

### Objectives

The aim of the study is to conduct a research intervention that will reveal the impact of the TPSR framework on students’ social outcomes in the Australian context. This research is significant because it will be the first time that TPSR has been tested in NSW high schools. The study will use a nonrandomized controlled trial design to determine feasibility and acceptability aspects.

## Methods

### Trial Registration

The study has been registered with the Australian New Zealand Clinical Trials Registry (ACTRN12624000858594p) and was approved on June 11, 2024.

### Ethical Considerations

This study has been approved by the University of Sydney Human Research Ethics Committee (2024/HE000496).

Students and parents will sign consent forms before taking part in this study. Students who do not want to participate will still attend PE lessons, but no data will be collected from them. Participants may withdraw from the intervention at any time by informing their facilitating teacher.

All participants will be deidentified, and no information about individual students or their school will be disclosed. Data will be password protected and securely stored by the principal investigator.

No compensation will be offered to participants or participating schools.

### Study Design

The protocol was developed according to the SPIRIT (Standard Protocol Items: Recommended for Interventional Trials) statement (Multimedia Appendix 1).

The study will use a nonrandomized controlled trial design with a qualitative evaluation to assess feasibility and acceptability. This method has been chosen for several reasons. First, one of the criticisms against the TPSR model is that it favors qualitative studies and has been underresearched using quantitative methodologies [11]. To address this, we will use both qualitative and quantitative methods in this study. The concern regarding the limited use of quantitative approaches was echoed in a systematic review on TPSR interventions that found that only 4 of the 22 included studies used exclusively quantitative methods, questioning the validity of the framework outside of qualitative research [26]. Studies have consistently called for greater use of quantitative analysis of the TPSR framework in research [10,11,26]. Furthermore, to obtain the most accurate representation of the effect of the framework in Australian schools, teachers, and students, this approach is deemed most suitable because it uses both qualitative and quantitative analysis and is able to factor in a complex landscape such as education [27]. In this study, data will be collected through student surveys, focus groups, teacher reflection journals, and the Tool for Assessing Responsibility-Based Education (TARE).

**Figure 1:**
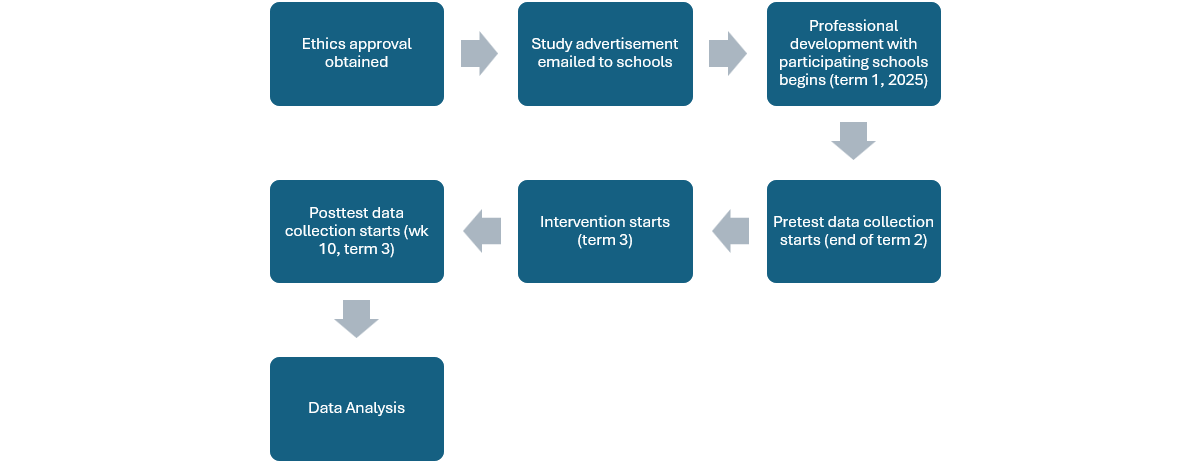
Study timeline.

We will recruit at least 6 classes from each school participating in the intervention, which will amount to approximately 3 classes in the control group and 3 in the experimental group (Figure 1).

Upon recruitment of the schools and selection of individual classes by each principal, researchers will collaborate with intervention facilitators to implement the TPSR framework during the intervention “season.” Table 1 presents example strategies that can be implemented in a TPSR study and will be shared with teachers during the co-design process.

A timeline of the intervention is presented in Table 2.

**Table 1:** Example strategies for implementing the teaching personal and social responsibility (TPSR) framework in physical education (PE) settings.

Levels	Explanation	Social outcomes in New South Wales PDHPE^a^ K-10 syllabus	Examples in PE setting
Respect for the rights and feelings of others	Demonstrating empathy for and understanding of other students’ feelings	Interact respectfully with others (“Rationale,” page 10) Stage 4: PD4-2^b^; PD4-3^c^; PD4-10^d^	Social “theme” of each lesson (eg, cooperation, teamwork, and leadership) Eye contact Not raising one’s voice during a confrontation
Effort and teamwork	Providing all students, regardless of skill level, the opportunity to be involved and included	Develop social skills (“Rationale,” page 10) Outcome: PD4-3; PD4-10	Team huddles Strategy time Giving everyone a turn
Self-direction and goal setting	Students’ role in the activity, their goals, and the goals of the team; collaboration between the two domains	Develop self-management skills with self-confident, socially responsible citizens (“Rationale,” page 10) Outcomes: PD4-2; PD4-9^e^	Discussing strategy Problem-solving Giving and embracing roles during games Students proposing modifications to the games
Helping and leadership	Demonstrating and developing leadership opportunities through play	Development of interpersonal skills (“Rationale,” page 10) Commitment to lead and promote healthy, safe, and active lives (“Values and attitudes,” page 13) Outcomes: PD4-2; PD4-3; PD4-10	Equipment roster Coaching and refereeing responsibilities Students acquire special roles independent of the teacher Student-teacher is chosen to instruct the group on the activities for the day instead of the teacher A new captain is chosen for each lesson
Transfer	How TPSR and the skills learned in lessons can be applied to other facets of life	Advocate for health, safety, and well-being of themselves and others in the community and beyond school (“Rationale,” page 10) Outcome: PD4-10	Reflective class discussion on how learning in this setting can be applied to other settings Student discussions in pairs

^a^PDHPE: personal development, health, and physical education.

^b^PD4-2: a student examines and demonstrates the role help-seeking strategies and behaviors play in supporting themselves and others.

^c^PD4-3: a student investigates effective strategies to promote inclusivity, equality, and respectful relationships.

^d^PD4-10: a student applies and refines interpersonal skills to assist themselves and others to interact respectfully and promote inclusion in a variety of groups or contexts.

^e^PD4-9: a student demonstrates self-management skills to effectively manage complex situations.

**Table 2:** Intervention timeline.

Terms and weeks		Tasks
**Term 2**		
	8	Preintervention testing PSRQ^a^ and PACES-PE^b^ surveys administered
	9	Back-up dates for surveys
	10	—^c^
**Term 3**		
	1 (athletics)	Zoom check-in (occurs every week) “Every name, every day” Class Goal HERO concept “Me time” “Good Game” at the conclusion of the lesson “Coaches and Players” in 3-week intervals
	2 (athletics)	TARE^d^ analysis
	3 (athletics)	—
	4 (athletics)	—
	5 (athletics)	TARE analysis
	6	—
	7	—
	8	TARE analysis
	9	—
	10	Postintervention testing PSRQ and PACES-PE surveys administered Focus groups completed
**Term 4**		
	Week 5	Follow-up

^a^PSRQ: Personal and Social Responsibility Questionnaire.

^b^PACES-PE: Physical Activity Enjoyment Scale–Physical Education version.

^c^Not applicable.

^d^TARE: Tool for Assessing Responsibility-Based Education.

### Outcomes

The primary outcomes addressed within the intervention are personal responsibility, social responsibility, and leadership, reflecting the levels of the TPSR framework. The secondary outcomes include enjoyment, feasibility, and acceptability. To obtain a comprehensive analysis of the TPSR framework within a school system, the proposed outcomes will provide valuable insights into how the framework influences student behavior and overall intervention effectiveness.

Baseline characteristics will include age, gender, boarding status (boarding vs nonboarding), school type, and SES (via postcode).

### Participants

The participants in this study will be students from years 5, 6, and 8. Each intervention group in the study will need to have 77 students and a matched control group of the same size to detect 2-tailed statistical significance (*P*<.05) with 80% power (1−β=0.8). Two schools in the western suburbs of Sydney have been recruited to participate in the study: one is a government high school, and the other is an independent K-12 school. They have been chosen through professional networks who expressed interest in the TPSR study. Students in NSW generally participate in 1 PE lesson per week, which is where the intervention will take place. PE lessons in NSW high schools are delivered to students from years 7 to 10 and focus on skills development, alongside one or two sport lessons focused on game play.

Consent will be obtained from parents, students, and the school principals in line with the ethics requirements of the University of Sydney. Participants’ personal information and school names will be deidentified in all publications arising from the study. Students and teachers will also be briefed on the intervention before study commencement and given the opportunity to ask questions. Participants will be allowed to withdraw from the intervention at any time. All data collected, including data from focus interviews, surveys, meeting minutes, and consent forms, will be stored securely and encrypted with a password. If parents request details of intervention results, a lay summary will be provided.

Fidelity and acceptability will be addressed through teacher expertise and training, intervention location, timetabling, and time spent on the intervention. Teachers will participate in a professional development program before starting the intervention. The purpose of this program is to familiarize teachers with the TPSR framework and to facilitate the co-design of strategies and activities to integrate the framework into programs. This approach also means that students do not have to build relationships with or adapt to outside facilitators. Furthermore, it does not incur additional funding for the school or research team. The professional development program will comprise a 3-hour, face-to-face learning block before the intervention and ongoing check-ins with teachers using Zoom (Zoom Video Communications, Inc) for the duration of the intervention.

The intention of the co-design process is to limit disruption to teachers and their practice as well as to create a mutually agreed-upon implementation of the TPSR framework in existing units. The co-design process allows both parties to benefit from sharing information that aims to improve currently established protocols and promotes sustainable practice within the school and with teachers [28]. Co-design principles can be applied at any stage of an intervention but may target three main components: (1) defining the problem, (2) designing a solution, and (3) implementation [29]. Throughout this intervention, teachers will work with researchers to identify social issues at their school; they will then work with researchers to apply TPSR principles to address these issues; and, finally, they will be involved in facilitating PE lessons that implement these principles with students. The co-design process aims to “share power” between the 2 parties taking part in the research with the mutual aim of improving outcomes [29]. This process will enable the research team and teachers to navigate the intervention in a collaborative manner, strengthening the planning process before intervention commencement.

### Measurement Tools

The Personal and Social Responsibility Questionnaire (PSRQ) will be used to measure the primary outcomes of the study: personal responsibility, social responsibility, and leadership. This questionnaire has been used in previous TPSR studies and has been deemed valid and reliable [30,31]. It consists of 14 items split into 2 categories (personal responsibility and social responsibility) [30]. Participants will complete the PSRQ in the pretest and posttest phases of the intervention and will be assured that there are no right or wrong responses and that their responses will remain anonymous. The TARE is an observational tool aligned with the TPSR model. The second part focuses on whether teachers have included elements such as modelling respect and setting expectations within their lessons [32]. The secondary outcomes of the study will be enjoyment, feasibility, and acceptability. These outcomes will be measured using the Physical Activity Enjoyment Scale–Physical Education version (PACES-PE), which is an 11-item measurement tool designed to measure enjoyment levels in adolescent children using a Likert scale and revised, student-friendly questions [33]. The PACES-PE has been used in PE-based studies previously and demonstrated positive results in female adolescent populations, particularly through the link between enjoyment and increased rates of physical activity [33].

### Recruitment Process

Two schools in NSW expressed interest in participating in the study during the 2025 school year. Upon being are recruited, a brief outlining the intervention design is emailed to principals, including the length of the intervention, its theoretical framework, and requirements for teachers and participating students. Meetings with participating schools will take place, and a co-design plan as well as professional development time will be organized. Consent forms and participation information statements will be distributed to the schools for collection from principals, teachers, students, and parents or guardians.

### Eligibility Criteria

Explicit eligibility criteria must be met for participation in the study. Students may participate if they (1) are enrolled in year 5, 6, or 8 at a participating independent or government secondary school in NSW; (2) have completed the pretest questionnaire; and (3) have written consent from parents or guardians.

### Intervention Design

This intervention will focus on providing opportunities for students to develop social skills in PE classes. Teachers will act as facilitators, and researchers will codevelop and adapt the intervention programs with them using a co-design process. A teacher professional development program will be administered before intervention commencement to ensure that teachers are familiar with the TPSR framework. Students will take part in focus groups before and after the intervention and will complete surveys measuring social outcomes. The researchers will be available for communication with teachers to maintain intervention integrity and to ensure that issues are addressed efficiently. Teachers will run their PE lessons with the TPSR framework embedded within their existing programs, minimizing additional workload. Examples of TPSR strategies that may be featured within lessons are provided in Table 1.

Before intervention commencement, students and teachers from the participating schools will complete preintervention testing, which will include surveys. Students at each school will then take part in the 10-week intervention and complete a postintervention test similar to the preintervention test. Focus groups will also be conducted during the posttest period. There will be a follow-up 5 weeks after the intervention. In line with the mandated requirements of the schools, students will participate in 1 PE lesson per week, which is standard practice in NSW schools. The duration of each lesson will depend on the school, with 45 to 60 minutes being typical in NSW schools. Social outcomes (personal responsibility and social responsibility) will be the primary focus of the intervention, as per the measurement tool.

### Data Analysis

This study will use a nonrandomized controlled trial design with a qualitative evaluation of feasibility and acceptability, integrating both qualitative and quantitative data to evaluate the impact of the TPSR intervention.

#### Quantitative Analysis

Quantitative data from the PSRQ, PACES-PE, and TPSR will be analyzed using SPSS software (version 30.0; IBM Corp). Internal consistency of the scales will be assessed using Cronbach α. The PSRQ will assess students’ self-perceived levels of personal and social responsibility, and the PACES-PE will assess enjoyment in PE. ANOVA will be conducted to compare mean scores before and after the intervention for both questionnaires. TARE data will be collected through lesson observations by the research team 3 times during the 10-week intervention period, and interrater reliability will be assessed using Cohen κ. Frequencies and mean scores of the TPSR strategies will be examined descriptively and across time to explore implementation fidelity. A repeated measures multivariate ANOVA will be performed to provide a more complex analysis of the findings and a more comprehensive view of how the intervention affects multiple variables simultaneously.

#### Qualitative Analysis

Focus group data will be collected from students to capture their experiences and perceptions of the TPSR program. Interviews will be analyzed using NVivo 15 (Lumivero) and the thematic analysis process developed by Clarke and Braun [34]. Representative quotes will be selected to exemplify key themes and bring student voice into the interpretation of the intervention’s impact [35].

### Fidelity

Fidelity measures are important in intervention studies because they reflect whether the intervention was implemented as intended [36]. Fidelity is also important when considering study replication and future work in related fields [36]. Ginsburg et al [36] argue that 3 common elements (ie, delivery, receipt, and enactment) are important in all fidelity models. This study aims to ensure that fidelity requirements are met through measures such as the professional development program for teachers, ensuring that they not only know and understand the TPSR framework but also know how to apply it in the intervention. This marks the co-design process as significant because researchers and teachers will work together to integrate TPSR elements into already existing PE programs. Fidelity measures will be gathered through both qualitative and quantitative methods, with students and teachers participating in focus groups before and after the intervention. Teachers will also complete a reflection journal at the conclusion of each lesson to assess the framework’s impact. This reflection journal will take <5 minutes to complete and will include the following questions:

1. “How would you rate student engagement with the responsibility themes today?” (1=not at all, 5=very highly)
2. “Did you observe any signs of student progress in any of the themes? Comment with dot points below”
3. “What was the most successful part of the lesson? Comment with dot points below”
4. “Any changes for next time?”

A member of the research team will conduct check-ins using Microsoft Teams or Zoom at the conclusion of weeks 1, 3, 5, 7, and 9 to ensure that teachers’ questions are answered and implement contingencies if needed or when responses to question 4 in the teacher reflection journal indicate that changes are warranted.

## Results

Recruitment for the study concluded in December 2024. Participation in the 10-week TPSR intervention will commenced in term 3 of the 2025 school year. Ethics approval was obtained in 2024 for the commencement of the study in 2025, and participating schools have been finalized. Data collection began in August, 2025, with the analysis scheduled for October 2025. Results are expected to be published in early 2026.

## Discussion

### Anticipated Findings

This study will be one of the first TPSR-based intervention studies conducted in NSW independent and government schools to evaluate outcomes in PE settings. Its evaluation aligns with the social outcomes articulated in the rationale and aims of the NSW PDHPE K-10 syllabus. The findings will contribute valuable data for future research, enabling comparisons of social interventions, both within Australian contexts and internationally, among similar cohorts. By extending existing research that focuses on psychomotor outcomes, this study aims to explore the broader impact of social interventions in PE, particularly in relation to responsibility, leadership, motivation, and engagement. If effective, the intervention has the potential to enhance students’ social development; relationships; and, by extension, academic performance. These improvements may also help address common barriers to participation in PE, including low self-esteem, bullying, and disengagement.

### Strengths and Limitations

One of the strengths of this study is that it represents the first TPSR investigation in secondary school settings in NSW, which may prompt further research into social outcomes in Australian schools. This is a significant strength due to the aforementioned benefits of fostering positive social behaviors. Furthermore, as one of the criticisms of past TPSR studies has been the lack of methodological diversity, this study will incorporate both qualitative and quantitative methods, thereby strengthening the literature in this field and addressing known limitations. In addition, positive findings may drastically transform school and classroom culture in the participating schools, potentially increasing participation and engagement in PE, an area that has faced criticism for low student involvement previously.

Predicted limitations of the study include the focus of the intervention solely on PE classes, which may limit the transfer of outcomes to other learning contexts. Moving forward, the TPSR framework may evolve to target the overall student experience across all key learning areas; however, given its current application only in PE, the effect of such transfer is difficult to gauge.

## Appendix

Multimedia Appendix 1 SPIRIT (Standard Protocol Items: Recommendations for Interventional Trials) guidelines.

## Abbreviations

AC: HPE: Australian Curriculum: Health and Physical Education
NSW: New South Wales
PACES-PE: Physical Activity Enjoyment Scale–Physical Education version
PDHPE: personal development, health, and physical education
PE: physical education
PSRQ: Personal and Social Responsibility Questionnaire
SES: socioeconomic status
SPIRIT: Standard Protocol Items: Recommendations for Interventional Trials
TARE: Tool for Assessing Responsibility-Based Education
TPSR: teaching personal and social responsibility
